# Iridescence as Camouflage

**DOI:** 10.1016/j.cub.2019.12.013

**Published:** 2020-02-03

**Authors:** Karin Kjernsmo, Heather M. Whitney, Nicholas E. Scott-Samuel, Joanna R. Hall, Henry Knowles, Laszlo Talas, Innes C. Cuthill

**Affiliations:** 1 School of Biological Sciences, University of Bristol, Bristol BS8 1TQ, UK; 2 School of Psychological Science, University of Bristol, Bristol BS8 1TU, UK

**Keywords:** anti-predator adaptation, iridescence, predation, protective coloration, camouflage, gloss, specular reflection, Sternocera aequisignata

## Abstract

Iridescence is a striking and taxonomically widespread form of animal coloration [1], but that its intense and varying hues could function as concealment [2] rather than signaling seems completely counterintuitive. Here, we show that the color changeability of biological iridescence, produced by multilayer cuticle reflectors in jewel beetle (*Sternocera aequisignata*) wing cases, provides effective protection against predation by birds. Importantly, we also show that the most likely mechanism to explain this increase in survival is camouflage and not some other protective function, such as aposematism. In two field experiments using wild birds and humans, we measured both the “survival” and direct detectability of iridescent and non-iridescent beetle models and demonstrated that the iridescent treatment fared best in both experiments. We also show that an increased level of specular reflection (gloss) of the leaf background leads to an increase in the survival of all targets and, for detectability by humans, enhances the camouflage effect of iridescence. The latter suggests that some prey, particularly iridescent ones, can increase their chance of survival against visually hunting predators even further by choosing glossier backgrounds. Our study is the first to present direct empirical evidence that biological iridescence can work as a form of camouflage, providing an adaptive explanation for its taxonomically widespread occurrence.

**Video Abstract:**

## Highlights

• Iridescence in prey can serve a counterintuitive function: concealment
• The effects of this protective function are further enhanced by glossy backgrounds
• Iridescence, even for signaling purposes, may be less costly than previously thought
• This newly discovered function may explain the widespread occurrence of iridescence

## Teaser

Kjernsmo et al. show that biological iridescence provides a survival benefit to prey by presenting iridescent and non-iridescent beetle models to birds and humans in a natural setting. They also show that the underlying mechanism for this is camouflage and that increased levels of specular reflection of the background further enhance this effect.

## Results and Discussion

Protective coloration in animals, including various forms of camouflage, aposematism, and mimicry, provides multiple ways for prey to escape predation [3, 4, 5]. However, the adaptive function of a vivid type of biological coloration, iridescence, is not fully understood [1]. Iridescence is generated by nanostructures that produce intensely chromatic colors that shift with changing angle of view or illumination [1, 6, 7, 8]. This variability can produce a striking visual appearance and make objects more conspicuous. Due to this, iridescence is often coupled with a signaling function [9, 10], frequently driven by sexual selection [1]. However, iridescence is also common in many monomorphic species. Although mutual ornamentation could be explained by sexual selection in some cases [11], and correlated selection remains a viable explanation for the female ornament in others [12], natural selection remains a possibility. Importantly, iridescence is found in non-reproductive stages, such as caterpillars and butterfly chrysalises [13, 14]. Here, sexual selection seems unlikely, although a warning role (aposematism) is certainly possible [15, 16]. Instead, the “father of camouflage,” Abbott Thayer [2], suggested that iridescence in many animals is actually camouflage, because the directionality of color in iridescent animals makes them appear “dissolved into many depths and distances” (p. 87). Thus, one of the first functional hypotheses for biological iridescence was that it conceals rather than reveals.

Despite being proposed more than a century ago, empirical support for Thayer’s theory of iridescence as camouflage has only appeared very recently [17, 18]. These studies confirmed that iridescence appeared to interfere with the ability of birds to successfully strike at simulated virtual prey [17] and with the ability of bees to identify a target shape [18]. Impeding prey recognition may therefore explain the evolution of iridescence in many monomorphic species. However, does biological iridescence actually provide a survival advantage against birds, likely to be one of the most important predators of iridescent insects, and, if so, what is the underlying mechanism: camouflage or aposematism?

**Figure 1:**
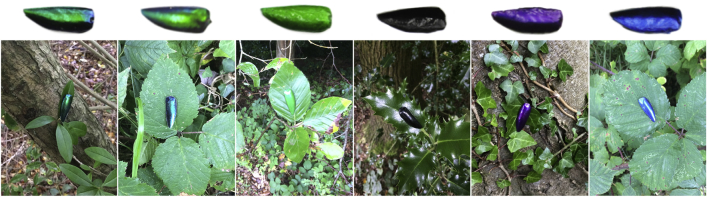
Prey Target Designs for All Six Treatments From left to right: iridescent on privet, static rainbow on bramble, green on beech, black on holly, purple on English ivy, and blue on bramble. Also illustrated in these images is the varying level of specular highlights (gloss) between the different backgrounds.

Using real, multilayer iridescent wing cases of the Asian jewel beetle (*Sternocera aequisignata*) and non-iridescent beetle wing case models as prey (Figure 1), we investigated these fundamental questions about the adaptive function of iridescence. In two separate field experiments, we tested the effects of iridescence on both survival and detectability of the prey targets. In experiment 1, we studied the survival of iridescent and non-iridescent targets against predation by wild birds in a natural setting. In experiment 2, we used humans as surrogate predators who searched for these targets in the same woodland location, enabling us to measure directly the detectability of iridescent and non-iridescent targets (see STAR Methods for full details). Controls included targets with the same reflectance peaks as seen in the iridescent targets (green, blue, and purple) and the same base color (black) and, to distinguish the benefits of having changeable colors from being multicolored but non-iridescent, targets wrapped with calibrated photos of the iridescent beetle cuticle (henceforth “static rainbow”; Figure 1).

For the bird experiment, we predicted that, if iridescence provides a survival advantage for prey, iridescent prey should have a higher probability of surviving than non-iridescent prey. For the human experiment, we predicted that, if the mechanism providing a survival benefit for iridescent prey was camouflage, the iridescent prey should be harder to detect. If, on the other hand, survival from the bird experiment is due to aposematism or neophobia, we predicted that the iridescent prey should be easy to detect in the human experiment. Finally, as the surface of these iridescent beetles also produces specular reflection of white light (hereafter, gloss, e.g., [19]), we also predicted an interactive effect of background gloss: iridescent beetles on glossy leaves should have a lower signal-to-noise ratio and thus be less detectable [20].

**Figure 2:**
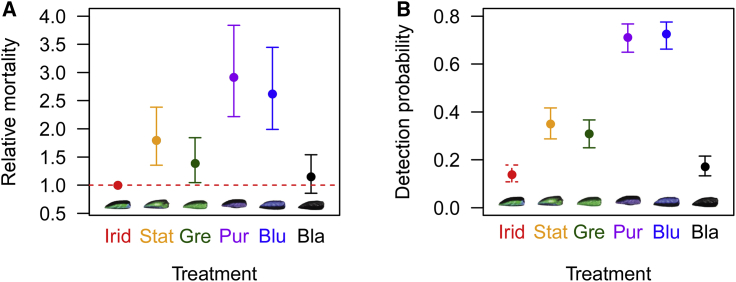
Avian Predation and Human Detection Data (A) Odds ratios (±95% confidence intervals [CIs]) from Cox mixed-model survival analysis comparing all treatments to the iridescent in the avian predation experiment. (B) Mean (±95% CI) probability of detecting targets for each treatment in the human detection experiment. In both experiments, the iridescent targets survived significantly better than all except the black treatment. Bla, black; Blu, blue; Gre, green; Irid, iridescent; Pur, purple; Stat, static rainbow. See also Figure S1A.

In the bird experiment, a total of 646 out of 886 targets (73%) showed evidence of avian predation; the rest were treated as censored in the survival analysis. Treatment affected relative mortality (Figure 2A; mixed-model Cox regression χ^2^ = 95.302; degrees of freedom [df] = 5; p < 0.001), with iridescent targets surviving better than all others except black (versus static rainbow, z = 4.05, p < 0.001; versus green, z = 2.26, p = 0.024; versus violet, z = 7.64, p < 0.001; versus blue, z = 6.87, p < 0.001; versus black, z = 0.93, p = 0.350). The human experiment mirrored the bird experiment (Figure 2B), with treatment affecting detection probability (generalized linear mixed model [GLMM] with binomial errors; χ^2^ = 699.13; df = 5; p < 0.001) and iridescent targets being less detectable than all others except black (versus static rainbow, z = 7.86, p < 0.001; versus green, z = 6.50, p < 0.001; versus purple, z = 17.00, p < 0.001; versus blue, z = 17.29, p < 0.001; versus black, z = 1.57, p = 0.118). For those targets that were detected, detection distance varied with treatment (GLMM with log-normal errors; F_5,1265_ = 27.54; p < 0.001). Iridescent targets were detected further away than black (t_1266_ = −2.72; p = 0.007) and at a similar distance to static rainbow targets (t_1266_ = 0.90; p = 0.367), but participants needed to be closer to them than green, purple, or blue to detect them (t_1265_ = 2.12, p = 0.033; t_1266_ = 3.61, p < 0.001; t_1266_ = 6.11, p < 0.001, respectively).

We next performed secondary analyses, including natural variation in background gloss as a covariate. In the bird experiment, there was no significant interaction between treatment and gloss (χ^2^ = 5.29; df = 5; p = 0.381), but average survival increased with the glossiness of the substrate (χ^2^ = 18.80; df = 1; p < 0.001) and again differed between treatments (χ^2^ = 96.70; df = 5; p < 0.001). Planned comparisons between the iridescent and five other treatments confirmed our primary analysis that the iridescent targets survived better than all treatments except black (versus static rainbow, z = 4.01, p < 0.001; versus green, z = 2.30, p = 0.022; versus violet, z = 7.68, p < 0.001; versus blue, z = 6.96, p < 0.001; versus black, z = 0.95, p = 0.340).

**Figure 3:**
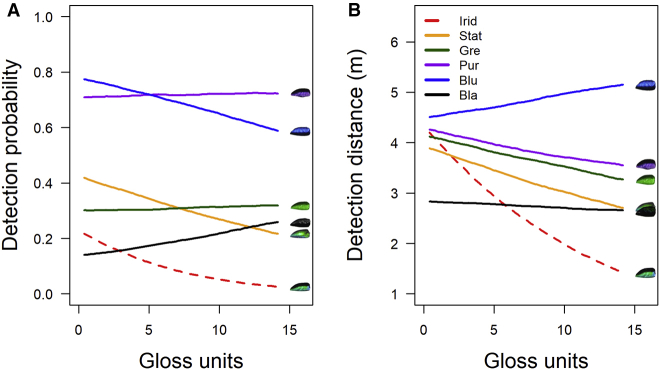
Results from the Human Detection Experiment Mean probability of detecting targets (A) and mean detection distance (B) as a function of gloss for each treatment. Lines are best fits from GLMMs. The iridescent targets became significantly more difficult to detect as substrate gloss increased, more so than other treatments. See also Figures S1C and S1D.

In the human experiment, there was a significant interaction between gloss and treatment for the probability of detecting a target (Figure 3A; χ^2^ = 16.58; df = 5; p = 0.005). The decrease in detection probability with increasing substrate gloss was significantly steeper for iridescent targets than green, purple, and black targets, but not static rainbow or blue (Table 1). When testing the relationship between gloss and treatment using GLMMs in which the iridescent treatment was contrasted against all the other treatments, the negative relationship with gloss was only significant for the iridescent treatment (Table 1). There was also a significant interaction between treatment and gloss for detection distance (Figure 3B; χ^2^ = 16.40; df = 5; p = 0.006). The decrease in detection distance with increasing substrate gloss was significantly steeper for iridescent targets than all others (Table 1). Indeed, the negative relationship with gloss was only significant for the iridescent and green treatments (Table 1).

**Table 1:** Human Experiment: Effect of Substrate Gloss on the Probability and Distance of Target Detection

Probability of Detection	Slope	t	p	Slope versus Irid (t)	Slope versus Irid (p)	Intercept	Intercept versus Irid (t)	Intercept versus Irid (p)
Iridescent	−0.15	−2.63	0.009	–	–	−1.09	–	–
Static	−0.05	−1.68	0.093	1.43	0.154	−0.34	3.11	0.002
Green	0.00	0.01	0.994	2.64	0.008	−0.88	1.13	0.258
Purple	0.00	0.05	0.958	2.04	0.042	0.88	6.26	<0.001
Blue	−0.07	−1.52	0.128	1.45	0.148	1.40	7.59	<0.001
Black	0.06	1.69	0.090	3.21	0.001	−1.70	−1.97	0.048

Distance								

Iridescent	−0.06	−2.25	0.027	–	–	1.44	–	–
Static	−0.01	−0.93	0.352	2.32	0.021	1.36	−1.05	0.293
Green	−0.03	−2.37	0.019	2.27	0.024	1.49	−0.17	0.863
Purple	−0.02	−1.08	0.280	2.11	0.035	1.44	−0.09	0.929
Blue	0.01	1.45	0.148	3.50	0.000	1.47	−0.06	0.956
Black	0.00	−0.28	0.784	2.87	0.004	0.97	−3.56	<0.001

Parameter estimates from GLMMs (binomial distribution, logit link) for detection probability and LMMs for detection distance (log transformed). All treatment slopes and intercepts are tested against the corresponding estimates for the iridescent treatment. Testing significance of individual intercepts is not of interest, only differences between treatment intercepts.

For humans, iridescent and black targets were least detectable on matte leaves, but on glossy leaves, iridescent targets outperformed black (Figure 3). In the bird experiment, the gloss^∗^target interaction was non-significant, but the trends were similar (Figure S1A), and the main effect of background gloss was still to reduce mortality. Taken together, the results suggest that an increase in the level of background specularity reduces detectability. This is plausibly because high specular reflectance acts as “visual noise,” decreasing the signal-to-noise ratio for target detection [20]. However, the disproportionate benefit to iridescent targets was not simply because they were glossier than other treatments (Figure S4B) or lighter in terms of diffuse reflectance (Figure S4A). The iridescent and static rainbow treatments had (by design) very similar lightness (and color) under diffuse illumination and yet very different survival. The black treatment was most different in lightness from the types of plant used in the study and much darker than any other treatment yet survived as well, and was as hard to detect, as the iridescent treatment on matte leaves. Previous studies on humans and birds have found that an increase of the visual complexity of the background makes objects harder to find [21, 22] and that some prey, in the presence of a predator, actively choose more visually complex backgrounds over those they simply match [23]. Specularity of the background is an under-researched factor affecting visual search that, given the effects we have demonstrated, merits attention. We predict that rainfall may have similar effects to leaf gloss.

With these experiments, we have clearly demonstrated an anti-predator function of iridescence: in both experiments, with wild birds or using humans as surrogate predators, the iridescent treatment fared best in terms of survival and probability of remaining undetected, particularly on glossy leaves. Importantly, the results from the human experiment clearly demonstrate that iridescence significantly lowers the probability of being detected, strongly suggesting camouflage as the underlying mechanism for the anti-predator function of iridescence. Non-visual factors that vary between plants and affect bird foraging (e.g., insect abundance) cannot account for the human detection results, and we note, the treatment differences in the bird experiment remain the same even when we limit the analysis to the single, commonest plant substrate, common ivy (*Hedera helix*; Figures S4B and S4C and accompanying analyses). It is noteworthy that the black treatment also fared well in both experiments, providing an adaptive explanation as to why so many insects in nature are black, in addition to any thermoregulatory benefits of melanization [24]. This is an important result, because it demonstrates that, in terms of visual perception of predators, there is no evident cost for prey to be iridescent compared to being black. Indeed, when adding the effect of background gloss, the iridescent treatment fared better than the black.

The reduced detectability and higher survival of iridescent targets compared to the static rainbow suggests that it is the *changeability* of the colors that is important for the protective function, not the presence of multiple colors on the same prey item. One possible explanation for this could be that iridescence, due to its changing colors, could serve as a form of dynamic disruptive camouflage [2, 25], producing inconsistent shape cues and interfering with feature binding [26]. Whatever the exact mechanism, our findings provide the first evidence to support Thayer’s more than century-old idea that biological iridescence can provide a survival benefit for prey through concealment. For sexually selected traits, the costs of iridescence may be lower than generally assumed. More generally, natural selection through predation could help explain the widespread occurrence of iridescence in many species of prey.

## STAR★Methods

### Key Resources Table

**Table:** 

REAGENT or RESOURCE	SOURCE	IDENTIFIER
**Deposited Data**		

Raw data	This paper	https://doi.org/10.5523/bris.388y3cip6r6mv25yccy5qum1l7

### Lead Contact and Materials Availability

Further information and requests for resources should be directed to and will be fulfilled by the Lead Contact, Karin Kjernsmo (karin.kjernsmo@bristol.ac.uk). This study did not generate unique reagents.

### Experimental Model and Subject Details

All human subjects (N = 36, 17 male and 19 female) that acted as ‘surrogate predators’ and performed the visual search task in experiment 2 gave their informed consent in line with the Declaration of Helsinki. The experiment was approved by the Faculty of Science Research Ethics Committee, University of Bristol.

### Method Details

#### Prey target design

Both experiments were conducted in Leigh Woods National Nature Reserve, North Somerset, UK (28 38.60 W, 518 27.8 0 N). We assessed the survival and detectability of beetle-like targets under predation by wild birds (experiment 1) and humans (experiment 2) in a natural setting by pinning real and artificial beetle wing cases (Figure 1) to leaves of various species of plants. To test whether iridescence provides a survival advantage compared to non-iridescent prey targets, we produced six different groups of prey targets, one iridescent and five controls. The iridescent targets were made from real jewel beetle (*Sternocera aequisignata*) wing cases. To design four of the controls, we used a hyperspectral camera (sensor: Hamamatsu Orca 03; unit: Resonon Pika UV; lens: UV Nikkor 105 mm) with a frequency range of 300-800 nm to identify the peak wavelengths reflected by the iridescent elytra of these jewel beetles, and tried to match these peaks as closely as possible using nail varnish to create single-colored, non-iridescent treatment groups (see Figures S2 and S3 for ternary plots (Maxwell color triangles) of all targets in bird and human color space, respectively). The measurements revealed peaks in the violet, blue and green end of the color spectrum. Finally, the ground color of the elytra was black, resulting in the following four control groups: black, violet, blue and green. The closest match to violet was ‘Vivid Violet’ (No 7, The Boots company PLC, UK); for blue was ‘661 Ocean Blue’ nail varnish (Maybelline, New York, USA); for green was a mix of ½ ‘Peacock Green’ nail varnish (No 7, The Boots Company, PLC, UK) and ½ of number ‘163 Metallic Green’ nail varnish (Kleancolor, Santa Fe Springs, California, USA); and for black was ‘Blackjack2′ nail varnish (Collection, BA14 0XB, UK).

To control for any differences in shape and size of the targets, we made epoxy resin copies of the real jewel beetle wing cases before painting them with their corresponding colors. To make the negative copies of the real jewel beetle wing cases, we used Elite HD+ Light Body silicone dental molds (Zhermack, Badia Polesine, Italy) with equal amounts of base and catalyst, and gently pushed jewel beetle wing cases (N = 100) into the mold mixture. After approximately 15 min, when the silicone dental mold mixture had hardened, the specimen was removed, leaving an inverted mold. The positive replicas of the jewel beetle wing cases were then made using ‘2-Ton’ epoxy resin. For each batch of 15 targets, we mixed 6.5 g resin + 6.5 g catalyst with 150 mg of “black” color pigment (L. Cornelissen and Son, London, UK). We then poured the mix into the molds, gently placed a pushpin in the middle and then left the targets overnight to harden (this process allowed the pushpin to stick to the target without using additional adhesives). The following day, the targets were painted on the top side with two coats of the corresponding nail varnish.

Finally, to test the key aspect of iridescence (i.e., the angular change in color) on detection and survival, we designed a control target that displayed all the colors of the iridescent wing cases, but without the angular change, hereafter referred to as the ‘static rainbow’ treatment. The static rainbow targets consisted of photographic copies of the real jewel beetle wing cases. To make these targets, we placed a random selection of jewel beetle wing cases (N = 100) outside in natural light and photographed them straight from above using a Nikon D90 DSLR camera. Photographs were taken such that the printed size of the beetles corresponded to the size of the real jewel beetles. Photographs contained an X-Rite ColorChecker Passport (X-Rite, Grand Rapids, MI, USA), which was used to calibrate the images according to the color standard using a purpose-written program (code available upon request) in MATLAB 2016b (MathWorks 2016). The photographs were then printed on glossy photo paper (Epson premium glossy photo paper inkjet S042155) using an Epson SureColor SC-P600 printer with the ‘premium gloss’ setting. To control for any differences in olfactory cues, specular reflection, or surface texture, and mask any UV differences, all targets from all six treatments were coated with a layer of transparent nail varnish (‘Super stay 3D gel effect plumping top coat’, Maybelline, New York). Targets did not differ in mean gloss (ANOVA: F_5,59_ = 1.22, p = 0.310; Figure S4B).

#### Experiment 1: Birds as predators

For this experiment, each prey target consisted of two components: an edible part consisting of frozen overnight, then thawed mealworms (*Tenebrio molitor*) and a non-edible beetle wing case from the six different treatments described above. To measure survival of the beetle targets against predation by wild birds [27], a total of 900 prey targets, N = 150 per treatment, were pinned at ground level (< 1.0 m) on to leaves of various species of plants such as English Ivy (*Hedera helix*), Bramble (*Rubus fruticosus*), Beech (*Fagus sylvatica*), Holly (*Ilex aquifolium*) and Hazel (*Corylus avellana*) in Leigh Woods National Nature Reserve. The targets were evenly distributed between treatments in ten replicate blocks, run in different areas of the wood on different dates between June and August 2017. Allocation to plant was random with respect to treatment and thus there were no differences in mean substrate gloss between treatments (F_5,880_ = 0.17, p = 0.972). Some targets broke during handling, but data were successfully recorded for 886 out of the 900 targets. We checked the survival of these targets at 2, 24, and 48 h. Predation by birds, which ate all or most of the mealworm, was scored as an event in the survival analysis. Predation by other animals included spiders, which sucked the fluids out and left a hollow exoskeleton, slugs, which left slime trails, and ants, which chopped off small pieces of the mealworm. Predation by animals other than birds, complete disappearance of a target, or survival to 48 h, were treated as censored values in the survival analysis.

#### Experiment 2: Humans as “predators”

While the setup in experiment 1 using wild birds as predators provides us with information about the direct survivability of iridescent versus non-iridescent targets, it does not provide a direct answer as to *why* the targets survived. If some targets survive better than others, this could still be due to two mutually exclusive mechanisms: either the birds failed to detect the targets, in which case the mechanism would be camouflage; or they detected the targets but did not attack them, in which case the lower predation rate could be explained by neophobia or aposematism [28]. To investigate the underlying mechanisms, camouflage or aversion, for any effects on survivability of our treatments, we conducted a similar study to experiment 1, but with humans as surrogate predators [22, 28]. Humans, as opposed to wild birds, can be given specific instructions before performing a search task, thus providing us with a direct measure of how difficult the targets were to detect.

The experiment was carried out between January and March 2018 in the same geographical area as the bird experiment. To assess the detectability of our targets, we placed a total of 174 targets (29 per treatment group) on leaves of the same mixed species, and at the same height, as in the bird predation experiment, in two separate blocks in Leigh Woods. Allocation to plant was random with respect to treatment and thus there was no differences in mean substrate gloss between treatments (F_5,168_ = 0.58, p = 0.716). Each participant (N = 36, 17 male and 19 female, with normal or corrected to normal vision) was equipped with a Leica DistoTM A6 laser rangefinder (Leica Geosystems, Heerbrugg, Switzerland) and asked to walk along one of the two transects in the woods and stop and point the laser rangefinder as soon as they detected a target. All targets were in plain sight and under 2 m from the path walked by the participants. Two measures were taken: whether a prey was detected or not and, if it was, the detection distance. Finally, to investigate whether specular reflection of the background had an impact on detection distance and detection probability, we also measured the level of gloss of each leaf that the targets were pinned to.

#### Effects of background gloss on detectability

It is possible that a glossier background could make it more difficult for an observer to detect a target: specular reflections might add to the visual complexity of the background, and background complexity reduces target detectability [21, 22]. Furthermore, as iridescent targets show modulation in intensity as well as hue, background specularities may act as distractors (in the technical sense used within the visual search literature: non-target objects similar to targets), so reducing the signal-to-noise ratio [20]. However, the effect of background gloss on the detectability of prey has never been tested before.

To investigate whether the level of glossiness of the background influenced the detectability of the targets, we collected all the leaves that each target had been pinned to from both experiments and measured their level of gloss using a ZGM 1120 Glossmeter (Zehntner Testing Instruments, Sissach, Switzerland) with the software ‘GlossTools’ (v.2.1, Zehntner Testing Instruments, Sissach, Switzerland). There is no single metric of perceived glossiness [29], but a major determinant is the relative amount of specular as opposed to diffuse reflectance, and this is what a glossmeter provides. The glossmeter measures gloss by recording the light reflected at 20, 60 and 85° away from the perpendicular to a surface. However, previous literature has shown that the recommended angle to use for small surfaces such as petals is 60° as it only requires a 4.7 × 2 mm aperture, and this is the measurement we use throughout [19]. The glossmeter was calibrated using the black polished glass standard that was supplied with the meter. Five readings were then taken from each leaf, and an average from the five readings was taken for each target. Leaf glossiness, in ‘gloss units’, was included as a continuous covariate in the secondary statistical analyses for both experiments.

### Quantification and Statistical Analysis

In both experiments, the primary analyses concern average detectability with respect to treatment. For experiment 1, the relative survival of the beetles was analyzed using a Mixed Effects Cox Model from the package coxme [30], with block as a random factor. For experiment 2, we used Generalized Linear Mixed Effects Models to analyze the log-transformed detection distance, and Generalized Linear Mixed Effects Models to analyze detection probability (binomial error, logit link) using functions lmer and glmer respectively in the package lme4 [31]. Block and subject were included as random effects. The model was re-levelled so that all planned, pairwise contrasts were conducted between the iridescent and other treatments. Secondary analyses were also carried out in a similar fashion, including gloss as a covariate; these are considered secondary analyses because background gloss was not manipulated. P values were calculated using the package lmerTest [32] using F-tests, with Satterthwaite degrees of freedom, to compare models with and without the factor in question. All analyses were conducted using the statistical software R v. 3.4.2 [33].

Average gloss differed between plant species (Figure S4C) and so could be a confounding factor in the main analyses. We could not include ‘plant’ as an additional factor in the analyses presented, because the frequency of the 19 different species was hugely unbalanced (across both human and bird experiments, from 1 birch leaf to 411 ivy). However, because ivy was both abundant in our sample and the leaves vary considerably in glossiness (the gloss values for ivy range from the 1.3 to the 98.6th percentile of all plant glosses in the study), we could repeat our analyses using only targets that were placed on ivy (39% of all samples).

In the bird experiment, as with the analysis of the complete dataset, there was no significant interaction between treatment and gloss (χ^2^ = 1.74, df = 5, p = 0.884) and survival differed between treatments (Figure S1B; χ^2^ = 49.70, df = 5, p < 0.001); the main effect of gloss was, however, not significant (χ^2^ = 0.16, df = 1, p = 0.691). Planned comparisons between the iridescent and five other treatments mirrored the analysis of the complete dataset: the iridescent targets survived better than all treatments except black (versus static rainbow, z = 2.93, p = 0.003; versus green, z = 2.53, p = 0.011; versus violet, z = 5.97, p < 0.001; versus blue, z = 4.58, p < 0.001; versus black, z = 0.93, p = 0.350).

In the human experiment, the interaction between gloss and treatment for the probability of detecting a target was not significant (Figure S1C; χ^2^ = 10.83, df = 5, p = 0.055). Despite the non-significance, it is relevant to explore whether the pattern of treatment differences is similar to that for the whole dataset. In separate GLMMs for each treatment, the negative relationship with gloss was only significant for the iridescent treatment (Iridescent: slope −0.30, X^2^ = 4.64, df = 1, p = 0.031; Static rainbow: slope −0.01, X^2^ = 0.03, df = 1, p = 0.862; Green: slope 0.01, X^2^ = 0.03, df = 1, p = 0.872; Purple: slope −0.08, X^2^ = 0.26, df = 1, p = 0.611; Blue: slope −0.5, X^2^ = 0.048, df = 1, p = 0.488; Black: slope 0.34, X^2^ = 3.27, df = 1, p = 0.071). The interaction between treatment and gloss for (log-transformed) detection distance was significant (Figure S1D; F_5,653_ = 2.30, df = 5, p = 0.043). The decrease in detection distance with increasing substrate gloss was significantly steeper for iridescent targets than all others except black (versus static rainbow, z = 2.85, p = 0.005; versus green, z = 2.49, p = 0.013; versus violet, z = 2.32, p = 0.021; versus blue, z = 2.84, p = 0.004; versus black, z = 1.89, p = 0.058).

### Data and Code Availability

The data from this study have been deposited in the University of Bristol data repository: https://doi.org/10.5523/bris.388y3cip6r6mv25yccy5qum1l7.

## Supplemental Information

Document S1. Figures S1–S4

Document S2. Article plus Supplemental Information

Video Abstract
