# Supplementary material for: Iridescence as Camouflage
Source: Curr Biol. 2020 Feb 3;30(3):551–555.e3. doi: 10.1016/j.cub.2019.12.013 (PMC6997887; doi:10.1016/j.cub.2019.12.013)
Supplement: Document S1. Figures S1–S4 [file mmc1.pdf]

**Current Biology, Volume *30***

## Supplemental Information

# Iridescence as Camouflage

**Karin Kjernsmo, Heather M. Whitney, Nicholas E. Scott-Samuel, Joanna R. Hall, Henry Knowles, Laszlo Talas, and Innes C. Cuthill**

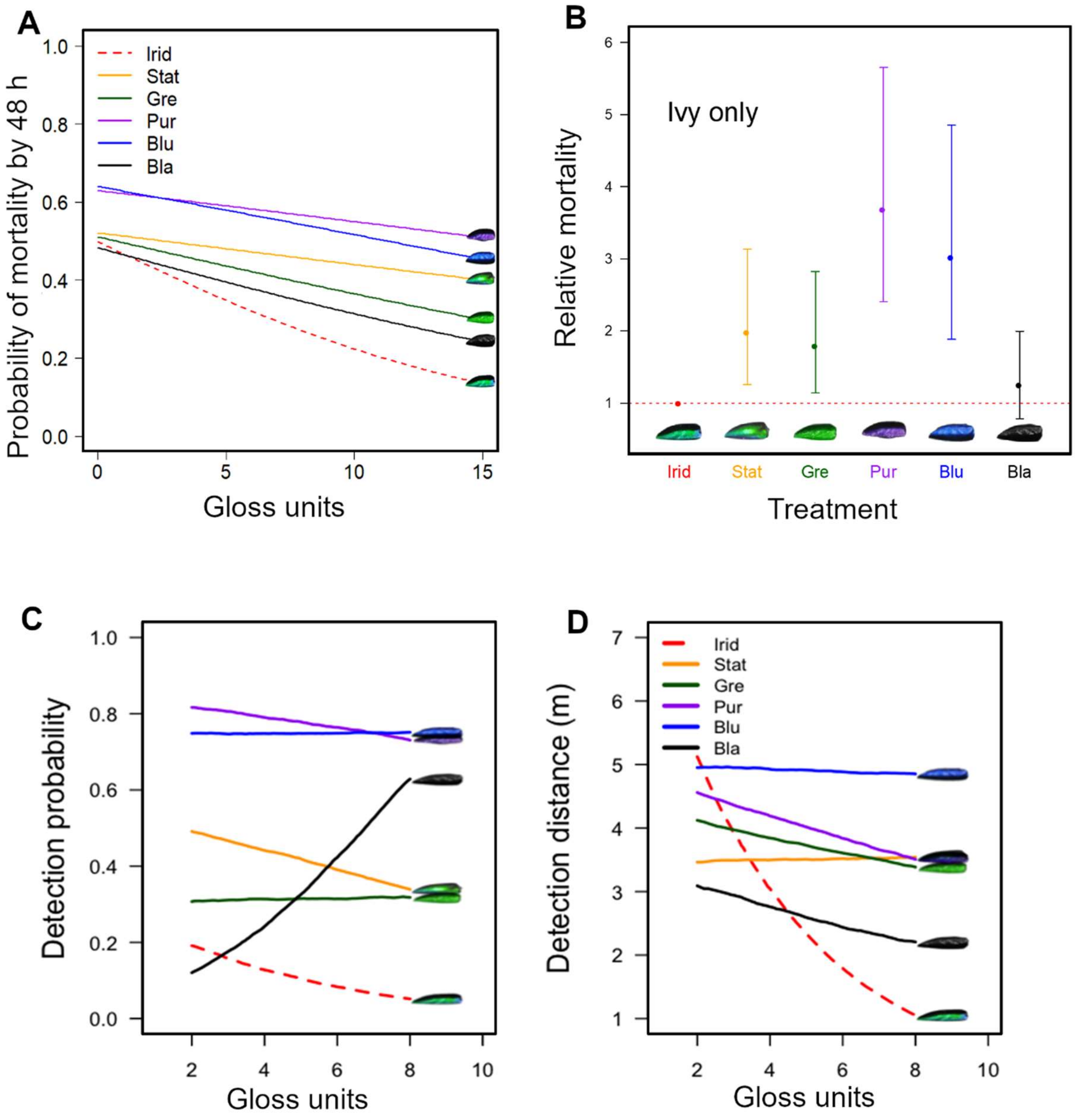


**Figure S1. Relative mortality, detection probability and detection distance for all targets. Related to Figures 2 and 3. A)** Probability of mortality by 48 h, as a function of background leaf gloss and target type, in the avian predation experiment. Survival estimates are from mixed model Cox regression. Although the treatment*gloss interaction was not significant (see main text), the iridescent targets tended to have higher survival on glossier leaves, consistent with the detection experiment with humans (Figure 3). Irid: iridescent; Stat: static rainbow; Gre: green; Pur: purple; Blu: blue; Bla: black. **B)** For targets placed on ivy leaves only: odds ratios (± 95% confidence intervals) from Cox mixed model survival analysis comparing all treatments to iridescent, in the avian predation experiment. Irid: iridescent; Stat: static rainbow; Gre: green; Pur: purple; Blu: blue; Bla: black. **C)** Mean probability of detecting targets from the human detection experiment and **D)** mean detection distance from the same experiment as a function of gloss, for each treatment. Lines are best fits from GLMMs. The iridescent targets became significantly more difficult to detect as substrate gloss increased, more so than other treatments. Irid: iridescent; Stat: static rainbow; Gre: green; Pur: purple; Blu: blue; Bla: black.

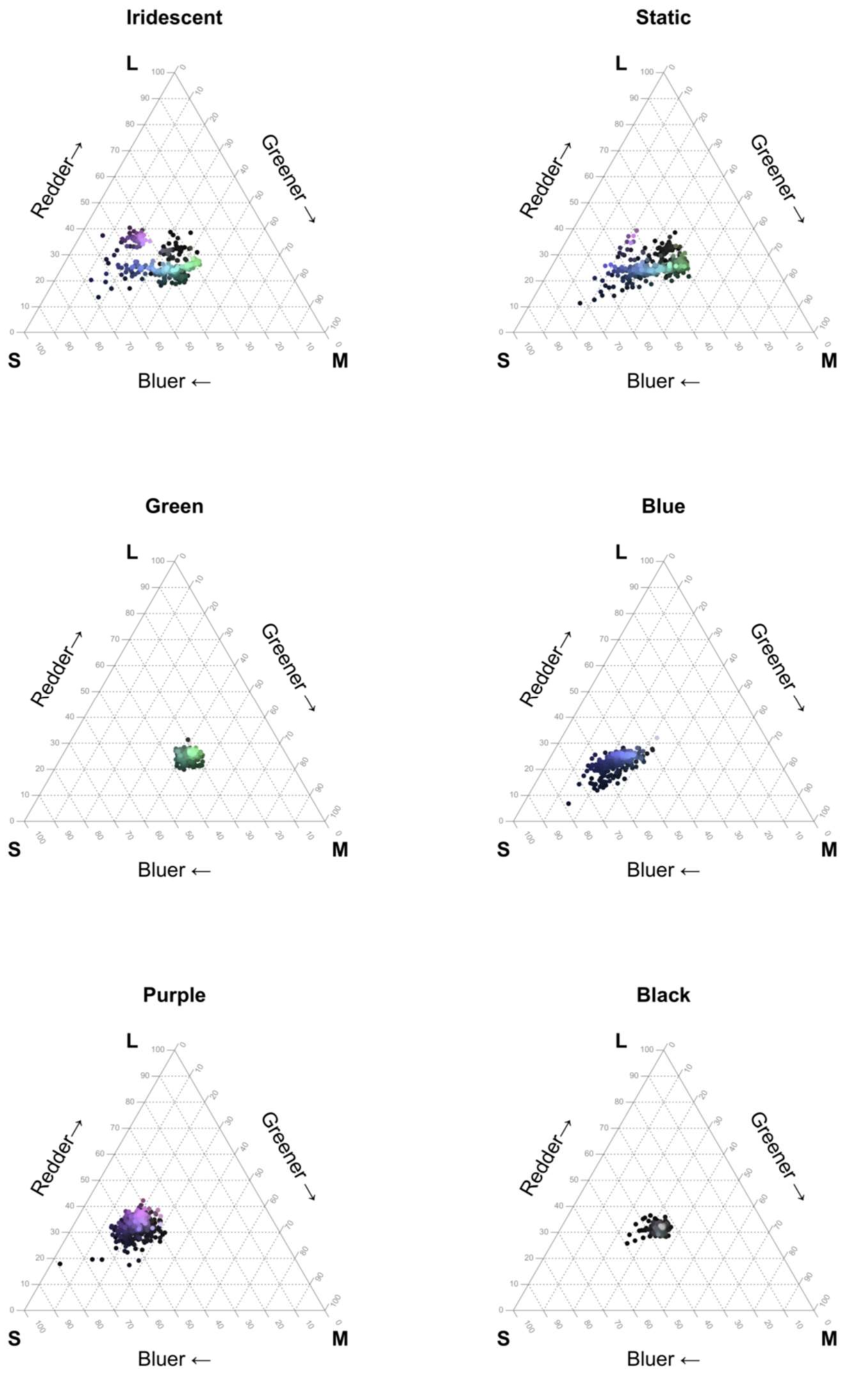


**Figure S2. Maxwell triangles (ternary plots [S1]) of the colours of the six target types in a simplified avian colour space**. **Related to section 'Prey target design' of the STAR Methods.** Points are 100 sampled pixels from photos of each of 10 targets, taken with a calibrated Nikon D3200 camera (Nikon Corp., Tokyo, Japan) and transformed to the photon captures of a blue tit (*Cyanistes caeruleus*) shortwave (S), mediumwave (M) and longwave (L) cones, using the methods in [20,26]. UV is omitted because of the minimal ultraviolet reflectance. The three axes in each plot are the percentage of photon catches by each of the three cone types, running from the respective apex (100%) to the mid-point of the side opposite (0%). The centre of a triangle is the achromatic locus (black-grey-white). Points are coloured according to human perception, for illustrative purposes only.

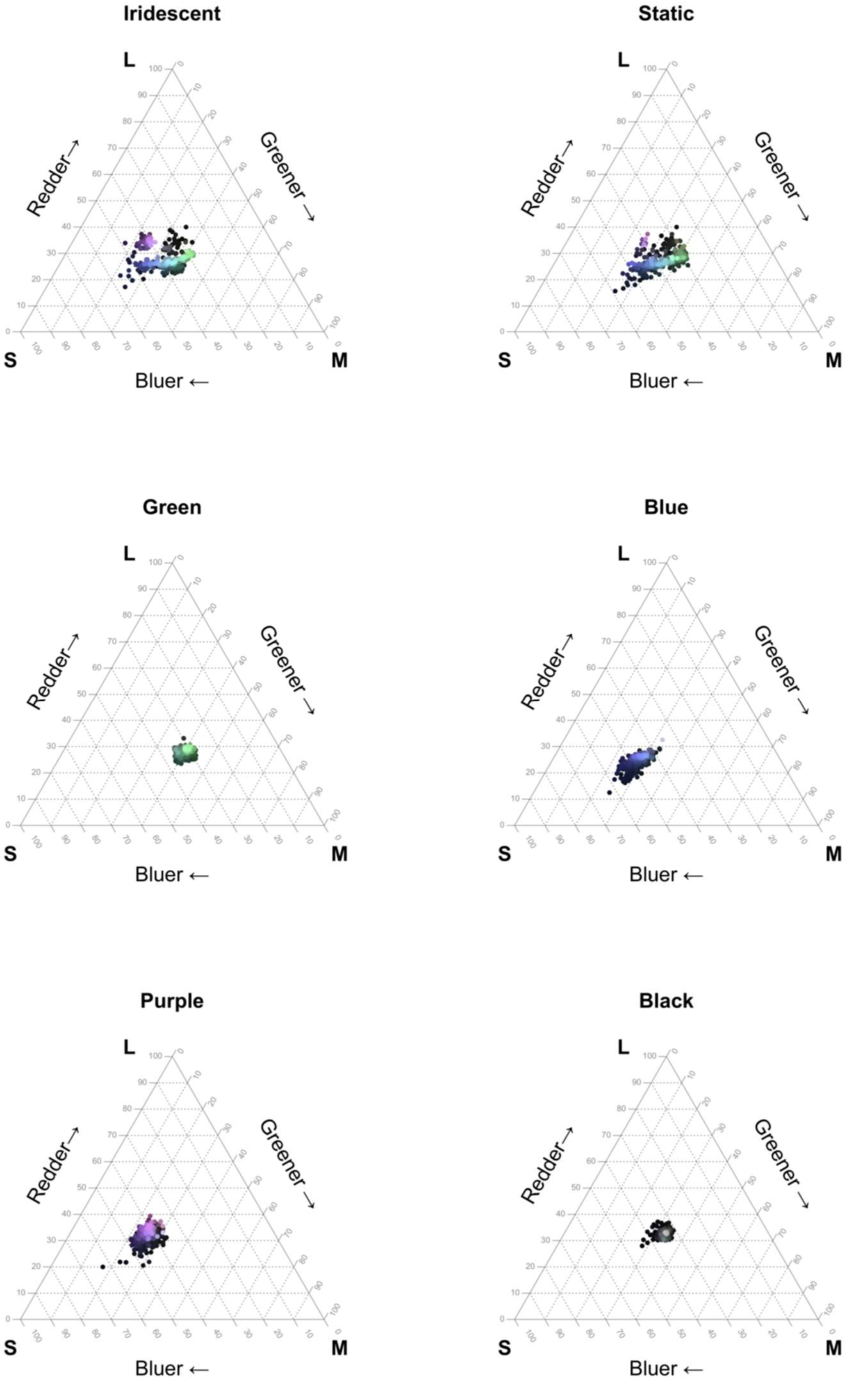


**Figure S3. Maxwell triangles (ternary plots [S1]) of the colours of the six target types in human cone colour space. Related to section 'Prey target design' of the STAR Methods.** Points are 100 sampled pixels from photos of each of 10 targets, taken with a calibrated Nikon D3200 camera (Nikon Corp., Tokyo, Japan). The three axes in each plot are the percentage of photon catches by each of the three cone types, running from the respective apex (100%) to the mid-point of the side opposite (0%). The centre of a triangle is the achromatic locus (black-grey-white). Points are coloured according to human perception.

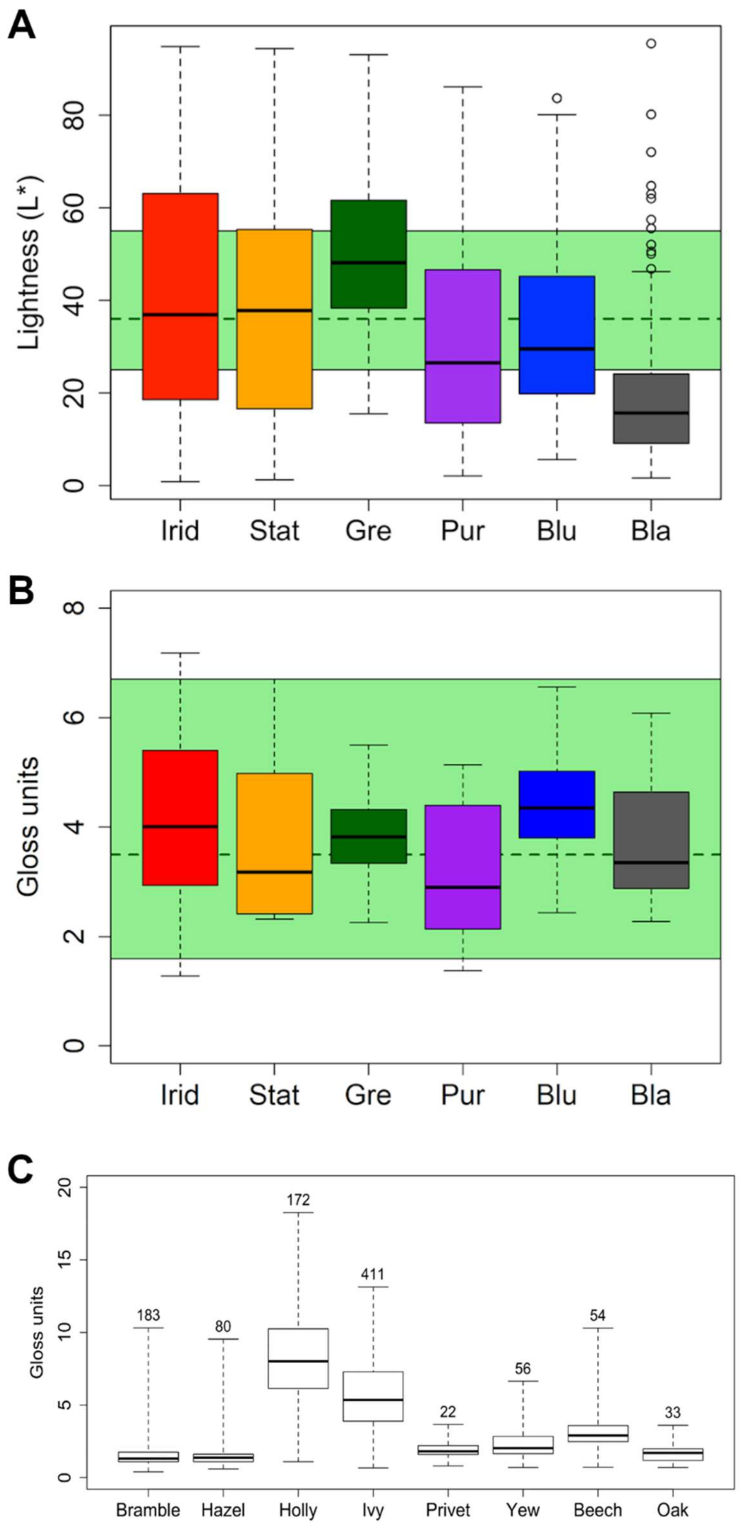


**Figure S4. Target and background lightness and gloss as well as the commonest species of plants comprising the background. Related to the Results and Discussion and to section 'Prey target design' of the STAR methods. A)** Achromatic lightness (L* in L*a*b* colour space [S2] of the data appearing in Figures S2 and S3, compared to the lightness of the main plant species in the study (shaded green area). **B)** The level of gloss for each treatment group (N = 10 per group), relative to

the level of gloss of the backgrounds used in the experiments (shaded green area). Boxes show the median ± IQR and whiskers show the min-max values. The top and bottom of the shaded green area denotes the IQR of the background (leaf) lightness and gloss, respectively, and the dashed lines show the medians. We have not plotted a measure of achromatic lightness for bird vision (double cone photon catch) because the correlation with human L is so high for these data ($r > 0.99$). Note also that the data for plant lightness (green shading in A) were from calibrated photographs of the same species used as backgrounds in the study, but not (unlike the gloss measures) the actual plants themselves. We have no such data. **C)** The commonest plant species to which targets were attached in the study, combining bird and human experiments. The 11 additional species, that each comprised 1% or fewer of the sample, have been omitted for clarity. Boxes show the median ± IQR and whiskers show the min-max values; numbers represent sample sizes. The Latin names of the plants are, left to right, *Rubus fruticosus, Corylus avellane, Ilex aquifolium, Hedera helix, Ligustrum* spp.*, Taxus baccata, Fagus sylvatica, Quercus robur.*

## Supplemental References


S1. Smith, M. R. (2017). Ternary: An R Package for Creating Ternary Plots. (https://ms609.github.io/Ternary).

S2. 1. CIE (1976). CIE Colorimetry - Part 4: 1976 L*a*b* Colour Space. Joint ISO/CIE Standard. ISO 11664-4:2008(E)/CIE S 014-4/E:2007. (Vienna, Austria: Commission Internationale de l'Eclairage).
